# Assessment of a new low-cost, PCR-based strategy for high-risk human papillomavirus DNA detection for cervical cancer prevention

**DOI:** 10.1186/s12879-019-4527-9

**Published:** 2019-10-15

**Authors:** Pedro Surriabre, Andrea Torrico, Tania Vargas, Fuantina Ugarte, Patricia Rodriguez, Véronique Fontaine

**Affiliations:** 1 Laboratorio de Virología, Facultad de Medicina, Universidad Mayor de San Simón, Cochabamba, Bolivia; 2 Unité de Microbiologie Pharmaceutique et Hygiène, Faculté de Pharmacie, Université Libre de Bruxelles (ULB), Brussels, Belgium; 3 CIES Salud Sexual y Reproductiva, Regional Cochabamba, Bolivia

**Keywords:** Human papillomavirus, HPV screening, PCR BSGP, pU, EIA, Collection devices

## Abstract

**Background:**

HPV test implementation as a primary screening tool has the potential to decrease cervical cancer incidence as shown by several studies around the world. However, in many low-resource settings, the HPV test introduction has been backed down mainly due to its price. In this study, we present a novel low-cost strategy involving simple devices and techniques for high-risk human papillomavirus (HR-HPV) detection. The analytical performance to detect HR-HPV infections of this novel strategy was assessed by comparing it with the Hybrid Capture 2 system (HC2), which is used as gold standard.

**Methods:**

Paired-cervical samples were collected from 541 women assisting to gynecological services in an outpatient clinic. One sample was transported in the Hybrid Capture Standard Transport Medium for HR-HPV detection by the HC2. The second sample was transported on glass slide for detection by PCR-based techniques (GP-EIA, BSGP-EIA and pU 1 M-L/2R).

**Results:**

The level of agreement between the PCR-based techniques and HC2 system was determined with the Cohen’s kappa value. The kappa values between HC2 and GP-EIA, BSGP-EIA and pU 1 M-L/2R were 0.71 (CI 95% 0.63–0.78), 0.78 (CI 95% 0.71–0.84) and 0.63 (CI 95% 0.55–0.72), respectively. However, when the results from both BSGP-EIA and pU 1 M-L/2R were combined, the level of agreement with HC2 was increased to 0.82 (CI 95% 0.76–0.88), reflecting a very good agreement between the two HR-HPV detection strategies. Furthermore, the sensitivity of both techniques combined was also increased compared to the BSGP-EIA (88.7% vs 77.4%) and the pU (88.7 vs 60.9%) without penalizing the specificity obtained with the BSGP-EIA (95.1% vs 96.9%) and the pU (95.1% vs 96.5%).

**Conclusions:**

This novel strategy, combining two PCR-based techniques for HR-HPV detection, could be useful for cervical cancer screening in self-collected samples in low-income countries.

## Background

Cervical cancer (CC) has the fourth highest rate for cancer incidence and mortality around the world. However, in many low-resource countries, CC becomes the first cause of female cancer and death [1]. Although the Papanicolaou (Pap) test has a low clinical sensitivity [2] to detect CC, it was for many decades the main diagnostic tool to prevent this disease. However, in less developed regions, due to limitations in trained personnel, the sensitivity of the cytology is low and the results are often either lost or given after long delays [3–6].

The discovery that an infection by the human papillomavirus (HPV) is a necessary cause for CC development has represented a milestone in the prevention of this pathology [7]. Twelve HPV genotypes have been classified as high risk (HR-HPV) namely 16, 18, 31, 33, 35, 39, 45, 51, 52, 56, 58 and 59; and 6 HPV genotypes were described as probably high risk (pHR-HPV) namely 26, 53, 66, 68 and 73 for CC development [8]. In this sense, the introduction of tests detecting HR-HPV genotypes (HPV tests) have improved the prevention of CC worldwide as they have been proved to be superior than the Pap test in terms of clinical sensitivity [2–5]. Indeed, many randomized controlled trials have proved the efficacy of HR-HPV-based screening programs starting at age 30 years [9]. One of the most widely used HR-HPV detection test is the Hybrid Capture® 2 (HC2) (Qiagen, USA) system which is based on the hybridization of viral DNA with RNA probes and antibodies that recognized the DNA-RNA hybrids. This technique has been clinically validated for detection of pre-cancerous and cancerous lesions of the cervix (CIN2+) and has been used as gold standard in many studies [4, 10].

Although most of the commercially available HPV tests have excellent clinical sensitivity and specificity values [11], they are unappropriated in large scale screening program in low resource settings mainly due to their high price. The use of low-cost devices to collect and transport cervical cells and of low-cost PCR-based techniques to detect HR-HPV infections are therefore suitable alternatives in developing countries. We have previously shown that vaginal cells, self-collected using a simple cotton swab and further self-smeared on a glass slide, can be valid sample for HR-HPV detection with PCR [12]. Lately, various PCR-based techniques have been developed to detect HR-HPV DNA but few allow for low-cost detection [13–18]. The PCR GP5+/6+, which targets the L1 region of the HPV genome, coupled with an enzymatic immunoassay (GP-EIA) has been extensively studied and used in different clinical settings, giving good results [19–21]. Nevertheless, some studies have reported that the original couple of GP5+/6+ primers underdetected certain HPV types (e.g. HPV 52) and multiple HPV infections [22–24]. Several modified primers and new amplification protocols were further proposed to replace the original primer couple method, e.g. BSGP primers [25], MGP primers [22], and GP touchdown protocol [26]. On the other hand, targeting the E6-E7 region represent an interesting option since this region remains present and highly expressed in high degree lesions and cancer [27, 28]. The pU1M-L/pU2R primer set hybridizing in this region offers this possibility [29, 30] allowing together with the GP primers the detection of HR-HPV infections in all cervical cancers.

In this study, we present a novel low-cost strategy that includes the use of a glass slide to transport cervical cells, and the use of two PCR to detect HR-HPV efficiently. The choice of these two PCR was based on the results obtained with PCR GP5+/6+ coupled with an EIA (GP-EIA), PCR BSGP5 + 6+ coupled with an EIA (BSGP-EIA) and PCR pU 1 M-L/2R (pU) compared to the results obtained by the HC2 system, used as gold standard for HR-HPV detection.

## Methods

### Study population and cervical sample collection

The Bio-ethical Committee of the “Universidad Mayor de San Simón” approved the study protocol (October 30th, 2014) and each participant signed an inform consent form before enrollment. A group of 541 women attending gynecological services were recruited in the outpatient clinic “CIES Salud Sexual y Reproductiva” in the city of Cochabamba, Bolivia. Paired-cervical samples were collected from each woman. One sample was taken for HR-HPV detection with the HC2 system (Qiagen, USA) using the Digene® HC2 DNA Collection Device (Qiagen, USA). A second sample was collected using a cyto-brush (Changjun Medical, China) and was smeared over a glass slide. Each slide was put inside a small cardboard box specifically designed for it. The box was then transported in a zippered storage bag to the laboratory. The patients received only the results obtained with the HC2 system.

### HR-HPV detection with the HC2 system

The HC2 system was used as gold standard in this study. This technique does not require DNA extraction and the HR-HPV DNA detection was performed exactly as indicated in the manufacturer’s protocol (Qiagen). The HC2 system allows the identification of 13 HPV genotypes: 16, 18, 31, 33, 35, 39, 45, 51, 52, 56, 58, 59 and 68.

### DNA preparation from samples transported in glass slides

Cells were detached from the glass slide using 400 μl of a solution containing 2,5% Chelex100 (Bio-Rad, USA) and 50 μg/ml of proteinase K (Promega, USA), placed in an Eppendorf tube and then vortexed for 15 s. Cell lysis was performed at 56 °C for 1 h and then the proteinase K was inactivated by heating at 95 °C for 10 min. Tubes were centrifuged at maximal speed and supernatants containing crude DNA were recovered. A quality control for DNA extraction was performed by a PCR using the primers PC04 (5′-CAACTTCATCCACGTTCACC-3′) and GH20 (5′-GAAGAGCCAAGGACAGGTAC-3′), amplifying a 260 bp fragment from the human β-globin gene [31]. Amplified fragments were visualized under UV light in a 2% agarose gel (Sigma-Aldrich, USA) incubated in 0.5 μg/ml ethidium bromide solution (Sigma-Aldrich, USA).

### PCR GP

General HPV DNA was detected using the GP couple of primers: GP5+ (5′-TTTGTTACTGTGGTAGATACTAC-3′) and GP6+ (5′-GAAAAATAAACTGTAAATCATATTC-3′). The GP6+ primer was biotynilated in order to perform an enzyme-linked assay afterwards. These primers amplify a 150 bp fragment from the L1 region of the HPV genome [32]. The PCR reaction mix contained 3.5 mM MgCl_2_, 0.2 mM dNTP mix, 1 μM of each primer, 0.625 U of GoTaq® G2 Hot Start Polymerase (Promega, USA). The total reaction volume was 25 μl with 5 μl of crude DNA extract. PCR was performed in a T100™ Thermal Cycler (Bio-Rad, USA). The amplification protocol used for this PCR was described by Fontaine et al. [33]. Amplified fragments were visualized under UV light in a 2% agarose gel (Sigma-Aldrich, USA) incubated in 0.5 μg/ml ethidium bromide solution (Sigma-Aldrich).

### PCR BSGP

HPV DNA was amplified using the modified set of primers BSGP5+/GP6+ (BS primers) which include 9 forward and 3 reverse primers [25]. The 3 reverse primers (BSGP6+) were biotinylated in order to perform an enzyme-linked assay afterwards. The PCR reactions were carried out in a total volume of 25 μl and performed in a T100™ Thermal Cycler (Bio-Rad, USA). The PCR reaction mix contains 3.5 mM MgCl_2_, 0.2 mM dNTP mix, 0.2 μM of each forward primer, 0.4 μM of each reverse primer, 0.625 U of GoTaq® G2 Hot Start Polymerase (Promega, USA) and 5 μl of crude DNA extract. The PCR conditions were the same described by Schmitt et al. [25].

### Enzyme-linked immunoassay (EIA)

Detection of HR-HPV DNA was performed using an EIA as described previously [34]. This assay was performed with the biotinylated PCR products generated from the biotinylated GP6+ or BSGP6+ primers. Oligo-probes for detection of 12 HR-HPV genotypes (16, 18, 31, 33, 35, 39, 45, 51, 52, 56, 58, 59) and 2 pHR-HPV (66 and 68) were used. We used a cut-off point which was the mean of the optical densities of 6 negative controls plus three times the standard deviation.

### PCR pU

HPV DNA was also amplified using the pU-1 M-L (5′-TGTCAAAAACCGTTGTGTCCAGAA GAAAA-3′) and pU-2R (5′-GAGCTGTCGCTTAATTGCTC-3′) pair of primers [30]. The PCR reaction mixture contains 3.5 mM MgCl_2_, 0.2 mM dNTP mix, 0.5 μM of each primer, 0.625 U of GoTaq® G2 Hot Start Polymerase (Promega, USA) and 5 μl of crude DNA extract. The PCR conditions were slightly modified from the original protocol. Briefly, 40 cycles of amplification were used including a denaturation step at 94 °C for 1 min, an annealing step at 55 °C for 45 s and an elongation step at 72 °C for 1 min.

### HPV genotyping and sequencing

To determine the HPV genotypes in samples with discordant results for HR-HPV detection, two techniques were used: INNO-LiPA HPV Genotyping Extra (Fujirebio, Japan) PCR products sequencing. The INNO-LiPA test allows the detection of 28 HPV genotypes (HPV 6, 11, 16, 18, 26, 31, 33, 35, 39, 40, 43, 44, 45, 51, 52, 53, 54, 56, 58, 59, 66, 68, 69, 70, 71, 73, 74, 82) by reverse hybridization of biotinylated amplicons with specific oligonucleotides probes which are immobilized on membrane strips. The test was performed exactly as indicated in the manufacturer’s manual. The HPV DNA amplification was done using the INNO-LiPA HPV Genotyping Extra Amp kit (Fujirebio, Japan) according to the manufacturer’s instructions, using the T100™ Thermal Cycler (Bio-Rad, USA) and thermocycler program as previously described [33]. HPV pU and GP PCR products purified using a GFX column (GE Healthcare, USA), according to the manufacturer’s instructions, were directly sequenced by the Mix2seq kit using the ABI 3730XL sequencer (Eurofins Genomics, Germany).

### Statistics

Statistical analyses were performed using SPSS software. Cohen’s kappa value was calculated to determine the level of agreement for HR-HPV detection results between the different techniques used in this study. Kappa values of < 0.20, 0.21 to 0.40, 0.41 to 0.60, 0.61 to 0.80 and 0.81 to 1.00 were considered poor, fair, moderate, good and very good agreement, respectively [35].

The sensitivity and specificity of the evaluated PCR tests were calculated considering the results obtained with the HC2 system as gold standard. A true positive (TP) correspond to a positive result yielded by the PCR test and the HC2 system. A true negative (TN) correspond to a negative result yielded by the PCR test and the HC2 system. A false positive (FP) correspond to a positive result yielded by the PCR test but negative for the HC2 system; and a false negative (FN) correspond to a negative result yielded by the PCR test but positive for the HC2 system. The sensitivity is calculated as follows: TP/(TP + FN). The specificity is calculated as follow: TN/(TN + FP) [36].

## Results

The presence of HR-HPV DNA was evaluated on 541 samples with four techniques: HC2 (gold standard), GP-EIA, BSGP-EIA and PCR pU 1 M-L/2R. The results from the PCR-based techniques were compared with the gold standard to determine the kappa value (degree of agreement) between these techniques. The HR-HPV detection results from all comparisons are summarized in Table 1. The kappa values, sensitivities and specificities from all the comparison are summarized in Table 2.

**Table 1:** Comparison of the HR-HPV detection results obtained by the HC2, GP-EIA, BSGP-EIA and pU

	GP-EIA		BSGP-EIA		pU		BSGP-EIA + pU		TOTAL
	Positive	Negative	Positive	Negative	Positive	Negative	Positive	Negative	
HC2 Positive	81	34	89	26	70	45	102	13	115
HC2 Negative	16	410	13	413	15	411	21	405	426
TOTAL	97	444	102	439	85	456	123	418	541

**Table 2:** Comparison of the kappa values, sensitivities and specificities obtained between the HC2 (gold standard) and the PCR-based techniques

PCR technique	Kappa value (CI 95%)	Sensitivity (CI 95%)	Specificity (CI 95%)
GP-EIA	0.71 (0.63–0.78)	70,4% (62,1 – 78,7)	96,2% (94,3 – 98,1)
BSGP-EIA	0.78 (0.71–0.84)	77,4% (69,7 – 85,1)	96,9% (95,3 – 98,5)
pU	0.63 (0.55–0.72)	60,9% (52,0 – 69,8)	96,5% (94,6 – 98,3)
BSGP-EIA + pU	0.82 (0.76–0.88)	88,7% (82,8 – 94,6)	95,1% (93,1 – 97,1)

We first performed HC2 and GP-EIA and compared the result agreement by calculating the kappa value between them. The kappa value obtained was 0.71 (CI 95% 0.63–0.78) which can be interpreted as a good level of agreement. The sensitivity and specificity obtained were 70.4% (CI95% 62.1–78.7) and 96.2% (CI95% 94.3–98.1) respectively (Table 2). Even though the kappa value could be considered high enough, we looked for an improved version of the GP primers to increase the sensitivity of our technique. In that sense, we incorporated the BS primers to improve the detection of HR-HPV infections. Indeed, the comparison between the HC2 and the BSGP-EIA gave us a better sensitivity (77.4% vs 70.4%) without penalizing the specificity (96.9% vs 96.2%) (Table 2). Moreover, the kappa value was increased to 0.78 which still means that the level of agreement between the HC2 and the BSGP-EIA is good.

As we were concerned to miss and under detect severe grade viral infections, we investigated the impact of using only or adding a PCR targeting the E6/E7 nucleotide sequences. The comparison between the HC2 and the PCR pU 1 M-L/2R gave us a kappa value of 0.63 (CI 95% 0.55–0.72), representing a good level of concordance between the two methods even though it was clearly lower than the kappa value observed between HC2 and the PCR-based-EIA. However, when we merged the results for HR-HPV detection obtained by the BSGP-EIA and the PCR pU (i.e. a sample is considered positive if BSGP-EIA or PCR pU test is positive and a sample is considered negative if both BSGP-EIA and PCR pU tests are negative) we observed an increased kappa value of 0.82 (CI 95% 0.76–0.88), representing a very good level of agreement between this new HR-HPV detection strategy and the commercial gold standard HC2 system. The sensitivity of both techniques combined (BSGP-EIA + pU) was also increased compared to the BSGP-EIA (88.7% vs 77.4%) and the pU (88.7 vs 60.9%) independently (Table 2). Furthermore, the specificity of the BSGP-EIA + pU combination was not considerably affected compared to the BSGP-EIA (95.1% vs 96.9%) and the pU (95.1% vs 96.5%).

The samples presenting discordant results between the HC2 system and the PCR-based techniques, were subjected to HPV genotyping and/or DNA sequence analysis. The results are summarized in Table 3. Among the 13 samples with a positive results for the HC2 system but negative for PCR pU and PCR BSGP-EIA, we found 6 single infections, 5 multiple infections and 2 false positive results. Eight samples had at least one type of the HR-HPV group. Only 2 samples contained infections by HPV16 or 18: one sample contained a co-infection by HPV16 and 18, and another sample contained a single infection by HPV18.

**Table 3:** HPV types found on samples having discordant results between BSGP-EIA + pU and the HC2 system

	Number of samples with			
	HC2 (+) BSGP-EIA (−) pU (−)	HC2 (−) BSGP-EIA (+) pU (−)	HC2 (−) BSGP-EIA (+) pU (+)	HC2 (−) BSGP-EIA (−) pU (+)
a single infection	6	2	4	1
multiples infections	5	3	3	7
at least one type of HR-HPV	8	5	7	7
a false positive result	2	1	0	0
Total	13	6	7	8
HPV type				
6			1	3
11				1
16	1^a^		2	3^b^
18	2^a^	1	2	3^b^
26	1			
31				1
33		1	1	
39	2			
44	1	2		
45	1	1		
51	2		1	3
52	2	3	4	2
53	2	1		
54	1			
56	1		1	1
58				1
66	1	1		1
67*				1
68	1	1		
70	2	1		
69/71**	1			1
74				1
82	1			1
85**	1			

(+): positive result

(−): negative result

* The HPV types 67 and 85 were identified by sequencing

** The INNO-LiPA version used do not distinguish between HPV69 et 71

^a^ A sample is infected by both HPV types 16 and 18

^b^ Two samples are infected by both HPV types 16 and 18

Finally, among the 21 samples that tested negative for HC2 but positive for either PCR pU or PCR BSGP-EIA, we found 7 single infections, 13 multiple infections and 1 false positive result. Nineteen samples had at least one type of the HR-HPV group. Nine samples contained infections by HPV 16 or 18: three samples contained a single infection by HPV16, 4 samples had a single infection by HPV18 and 2 samples presented a co-infection by HPV16 and 18. These results show that our PCR-based strategy is able to detect more infections by the most oncogenic HPV types (16 and 18) than the HC2 system.

## Discussion

In this study, we present a low-cost strategy to detect HR-HPV for cervical cancer screening in low-resource settings. We searched for simple robust and cheap method to extract cell DNA and detect specifically HR-HPV DNA. The efficiency of this strategy to detect infections by HR-HPV types was evaluated by comparing it with the HC2 system.

Our laboratory has recently presented evidence supporting the use of a simple cervical brush and a glass slide to efficiently collect and transport cervical cells sample for HPV DNA detection by PCR [12]. Furthermore, the DNA preparation protocol that we used here is very simple and requires only 2 chemical reagents: Chelex-100 and proteinase K. This protocol was successfully used before for DNA recovery and detection by PCR [37]. It is neither time-consuming nor expensive to perform since the amount of reagents used per sample is very low. Furthermore, the percentage of successful DNA extractions using our protocol was near 100% (data not shown).

Concerning the HR-HPV detection, we chose to perform PCR, as it is a simple, sensitive, effective and low-cost technique. The GP-EIA method has been one of the most used PCR-based technique to detect HR-HPV DNA and compared with the HC2 system it gave good results to detect CIN2+ lesions [19]. However, many studies reported sensitivity problems with some HPV genotypes using the GP5+/6+ primers set, especially in presence of multiple infections [24, 38, 39]. Modifications of the GP primers or of the amplification protocol were proposed to circumvent this obstacle [22, 25, 26]. The modified BS primers were designed by Schmitt et al. to improve the GP system. This set of primers include 9 forward (BSGP5+) and 3 reverse primers (BSGP6+) which are biotinylated [25]. The BS primers were originally designed to be used with the LUMINEX technology, and to our knowledge, this is the first time that they were used coupled with an EIA. We have chosen to use the modified BS primers since we obtained with them a better agreement with the HC2 than the original set of GP primers. Indeed, the kappa values obtained with the original GP primers and with the BS primers were 0.70 and 0.78 respectively (Table 2). Furthermore, the sensitivity was increased using the BSGP primers without penalizing the specificity.

Another concern in the GP system is that it amplifies the L1 region of the viral genome. This region is thought to be disrupted when the viral DNA integrates into the host genome. The presence of only a viral integrated DNA form in cervical cells, in the absence of episomal forms, could occur in cervical cells, especially in high degree lesions. However, it is believed to be a rare event [40, 41]. As this could lead to some false negative results using only L1 targeting PCR, we decide to circumvent somehow this obstacle by including the PCR pU in our study. The pU primers were designed to amplify the viral DNA in the regions E6-E7 and they have been successfully evaluated to detect HR-HPV infections in invasive cancer samples [29, 30]. The first studies published about the PCR pU reported that it could amplify the DNA from at least 6 HR-HPV genotypes, namely 16, 18, 31, 52 and 56 [29]. However, the identity of all HPV genotypes detected with this PCR is still not clear. Nevertheless, the agreement value that we obtained between the PCR pU and the HC2 system was still categorized as good (κ = 0.63).

Several studies have performed a comparison between the GP-EIA technique and the HC2 system for HR-HPV detection. Kappa values in these studies range from 0.59 to 0.74 [20, 42–44], in agreement with our results showing a kappa value of 0.71. Furthermore, a recent meta-analysis, that compiled information from different studies that evaluated commercially techniques for HR-HPV detection with the HC2, reported kappa values ranging from 0.60 to 0.79 for those comparisons [45]. Considering all these reported agreement values reported in the scientific literature, we can be confident about the values obtained with our PCR techniques. Furthermore, when we based the HR-HPV DNA detection on two PCR-based techniques, BSGP-EIA and pU, the combined results are in a very good agreement with HC2 (κ = 0.82), giving higher kappa score than most comparisons between various commercial techniques and the HC2 standard. Both techniques BSGP-EIA and pU could be considered as complementary since the combination of both increase the sensitivity obtained with each technique separately without penalizing the specificity.

Furthermore, we genotyped and/or sequenced the samples with discordant results between the HC2 system and our PCR-based techniques in order to analyze which kind of HPV infections were missed or over-detected by our strategy compared to the reference technique. It is noteworthy that among the 13 HPV-infected samples that were missed by our PCR strategy, there were only 2 infections by HPV16 or 18 (15%) which are the most oncogenic genotypes. On the other hand, 43% (9/21) of the samples that tested positive by our PCR strategy but negative for HC2 system contained infections by either HPV16 or 18. These results altogether show that our PCR-based strategy is missing fewer infections by HPV16 and 18, which is an important key feature for a HPV test in a primary screening program.

## Conclusions

We present analytical evidence supporting the use of a low-cost strategy involving simple devices and techniques for cervical sample collection and transport, and for HR-HPV DNA detection with two PCR-based techniques used together (BSGP-EIA and pU).

This novel strategy could represent an interesting option to screen rural population for cervical cancer specially by self-collection of samples. Indeed, self-sampling with our simple devices (cotton swab and glass slide) seems well accepted among the Bolivian population, especially in rural areas, and could therefore increase the screening coverage rate of this population, as suggested by our preliminary study [46]. Furthermore, a clinical validation of the use of our PCR-based strategy in self-collected samples will be presented in a forthcoming publication.

## Data Availability

The datasets generated and/or analyzed during the current study are available in the Zenodo repository, 10.5281/zenodo.3445396.

## Abbreviations

bp: Base pair
CC: Cervical cancer
CIN2+: Cervical intraepithelial neoplasia of grade 2 or 1
DNA: Deoxyribonucleic acid
EIA: Enzyme immuno-assay
HC2: Hybrid Capture 2
HPV: Human papillomavirus
HR-HPV: High-risk types of human papillomavirus
Pap: Papanicolaou
PCR: Polymerase chain reaction
pHR-HPV: Probable high-risk types of human papillomavirus
RNA: Ribonucleic acid
